# Screening of preterm labor in Yazd city: transvaginal ultrasound assessment of the length of cervix in the second trimester

**Published:** 2013-04

**Authors:** Maryam Dalili, Mohamad Ali Karimzadeh Meybodi, Mohamad Ghaforzadeh, Tahmineh Farajkhoda, Hossein Molavi-e Vardanjani

**Affiliations:** 1 *Department of Obstetrics and Gynecology, Afzalipour Hospital, Kerman University of Medical Sciences, Kerman, Iran.*; 2 *Depatment of Obstetrics and Gynecology, Research and Clinical Center for Infertility, Shahid Sadoughi University of Medical Sciences, Yazd, Iran.*; 3 *Department of Midwifery, School of Nursing and Midwifery, Shahid Sadoughi University of Medical Sciences, Yazd, Iran. *; 4 *Research Center for Modeling in Health, Institute for Futures Studies in Health, Kerman University of Medical Sciences, Kerman, Iran.*

**Keywords:** *Early detection*, *Obstetric labor*, *Ultrasonography*, *Cervix uteri*, *Iran*

## Abstract

**Background: **Spontaneous preterm labor is one of the common obstetrics problems causing several physical, psychological and economical outcomes. Although due to these outcomes and the efficacy of cares for decreasing them, preterm labor screening is cost-effective and it is still one of the challenging issues in obstetrics.

**Objective:** In this study preterm labor screening by using cervical transvaginal sonography was evaluated.

**Materials and Methods: **This observational cohort study was performed in Yazd, Iran. Samples were selected from pregnant women at gestational age of 21-24 weeks who had single live fetus and referred to the obstetrics clinics of two selected hospitals in Yazd. Gestational age was estimated based on the sonography of the first trimester and cervical length measured by transvaginal sonography. Data analysis was done by using t and x^2^ test as well as ANOVA. Statistical significant level was considered as p<0.05.

**Results:** From 450 participants, 47 cases had preterm labor and 6 cases had positive funneling. Mean age of women with term labor was 26.09±4.13 years and that of women with preterm labor was 26.7±3.51 years (p=0.334). Duration of pregnancy and cervical length significantly differed between women with and without funneling (p=0.001). The sensitivity and specificity of screening based on cervical length of 25mm were 55.5% (50.9-60.1%) and 93.6% (91.2-96%) respectively.

**Conclusion:** Based on the results of the present study, transvaginal ultrasound assessment of cervical length in low risk women has an acceptable reliability for screening of preterm labor.

## Introduction

Preterm labor is one of the common obstetrics problems with an annual worldwide incidence rate of 13,000,000 cases (1). It is the major cause of prenatal mortality and is usually associated with recognized maternal and neonatal outcomes (2-7). Moreover, preterm labor imposes psychological and socio-economical pressures on both neonate’s parents and the society (8, 9). Spontaneous preterm delivery (SPTD) accounts for approximately 72% of preterm deliveries and several factors can increase the risk of this type of preterm delivery. Some of them are early threatened abortion in the current pregnancy, genetic factors, demographic features, behavior features such as cigarette smoking, low maternal weight gain during pregnancy, consumption of illicit drugs, pregnancy in extremes of reproductive life, short stature, vitamin C deficiency, prolonged walking and standing, exhausting activities and psycho-physical stresses (6, 10-17). Early diagnosis of high risk pregnancies for preterm labor and its outcomes has significant role in decreasing the incidence and mortality rates (5). 

According to the previous studies, the risk scoring system for predicting preterm birth developed by Papiernnik and its modified version by Creasy have not been efficient in the diagnosis of several cases leading to preterm labor (2, 17). There are different techniques for the diagnosis of preterm labor (18-21). Khani *et al* have used β-HCG level in cervicovaginal secretions as an indicator for the risk of preterm labor. They studied 354 high risk pregnant women in 24-28 weeks of gestation and have determined cervicovaginal β-HCG level of 30mu as cut off point. They asserted that this factor can be beneficial in predicting preterm labor (22). Considering the positive relationship between short cervical length and increased rate of preterm labor incidence, measurement of cervical length can be suggested as an efficient way for predicting preterm labor (3, 4, 21, 23-25). 

In comparison to other techniques, in transvaginal ultrasound assessment the quality and details of cervical canal can be observed better due to the closeness of the probe to the cervix (5, 25). Although the cost effectiveness and non-invasiveness of transvaginal sonography have been proved in several studies, in most of them further studies have been recommended (3, 5, 18, 26, 27). For example, Mahshidian *et al* have used cervical sonography parameters for predicting preterm labor in high risk women. In the mentioned study, cervical length, funneling and glands were compared between two groups of women with and without preterm labor. In their observational study, on 200 pregnant women with one of the risk factors of preterm labor they concluded that sensitivity and specificity of cervical length <18mm for preterm labor in gestational age of 35^th ^wk or less are 25% and 99% respectively. The same values for cervical funneling were found to be 50% and 91.8% respectively. Finally, the researchers suggested further studies with greater sample size (21).

Considering the results of the mentioned study and also genetic and environmental differences of societies, evaluation of the results of studies in other countries for their efficacy in our country seems to be necessary (28). Therefore, the present study was performed with the aim of evaluating the efficacy of measuring cervical length and funneling by transvaginal sonography at gestational age of 21-24 wk in screening of preterm labor in Yazd city, Iran.

## Materials and methods

This study was an observational prospective cohort project in Yazd city. The permission was obtained from the Vice Chancellor of Treatment and Ethics Committee, Yazd University of Medical Sciences. In the obstetrics clinics of Shahid Sadooghi and Madar Hospitals in Yazd, participants were selected from the aimed population. Inclusion criteria were having gestational age of 21-24 wk, single pregnancy and live fetus. 

Those with special condition disturbing the results like induced preterm labor due to sever preeclampsia, HELLP syndrome, severe IUGR (intra uterine growth restriction), abruption, fetal distress were excluded. Sequential sampling was performed and sample size, based on Carley *et al* study and considering 80% response rate had been determined 450 pregnant women (29). After considering the conditions of women referred to the clinics based on the inclusion criteria, the aims and process of study were explained to them and they were asked to give their consent for participating in the study. It should be mentioned that in studying the inclusion criteria, gestational age was estimated based on the last menstrual period (LMP) and its agreement with the result of sonography in the first trimester.

In the next stage, data related to age, job, educational level, gestational age, history of previous preterm labor, cerclage and curettage obtained in face to face interview and also extracted from the patients’ medical files, were recorded in the previously prepared forms. Then participants underwent transvaginal sonography at 21-24 wk (one time) and their cervical length or the presence of funneling was evaluated. Transvaginal sonography was performed after bladder emptying in dorsal lithotomy position and by 7.5 MHZ transducer. 

In order to observe sagittal cervical profile, probe was placed in anterior vaginal fornix and for observing internal and external cervical os, it was slightly moved and the distance between the echodensity of external os and V-shaped region of internal os was measured. Cases with the extrusion of fetal membranes into the cervical canal in a Y shape were considered as positive funneling. After recording data and performing transvaginal sonography, follow up for determining the exact time of delivery was started and time of delivery was recorded for all subjects. The study had no cost for participants and the secrecy of their data was considered.

**Statistical analysis**

Data analysis was done through SPSS11.5 and STATA 8 software packages by using t and x^2^ tests as well as ANOVA. In order to estimate sensitivity (SN), specificity (SP), positive predictive value (PPV) and negative predictive value (NPV) and their confidence range, SPSS software was used. Statistical significant level was considered as p<0.05.

## Results

A total of 450 women from 508 eligible participants were scheduled in this study. Mean age of participants was 26.16±4.07 years with the range of 15-45 years. From all, 53 cases had history of previous curettage and 42 cases reported history of preterm labor. In regard to the educational level, 101 cases were illiterate, 277 cases had high school Diploma or lower and 70 cases had higher degrees. In total, 404 women did not work out, 26 had low physical activity, 16 had difficult jobs and 4 women did not report their jobs. Considering gestational age <37wk as a criterion for preterm labor, 47 women had preterm labor and according to the results of transvaginal sonography, 6 cases were positive for funneling. 

Mean age of subjects with term labor was 26.09±4.13 years and that of those with spontaneous preterm labor was 26.7±3.51 years that shows no significant difference (p=0.334). Mean cervical length of whole participants was 35.25±6.87mm and it showed significant difference (p=0.001) in the two groups of term (36.53±5.81mm) and preterm (24.31±5.33mm). Statistical analysis showed significant relationship between the cervical length and duration of pregnancy (p=0.0001). Table I shows duration of pregnancy, the number of preterm labors and age distribution of women based on cervical length classification.

As it is seen, mean cervical length in women with history of preterm labor is 28.76±4.09mm that shows significant difference as compared to 36.8±6.7mm in those without history of preterm labor (p=0.0001). According to the results, two groups of term and preterm labor had no significant difference in regard to the educational level (p=0.18), but they had significant difference in regard to job (p=0.001). Mean pregnancy duration and mean cervical length in women with positive funneling were respectively 32±2.5wk and 15.0±2.1mm, while the same values for women with negative funneling were respectively 38.8±1.3wk and 35.5±6.5mm that is evident of significant difference between the two groups (p=0.001). Table II shows the results of evaluation of SPTD screening based on the cervical length measured through transvaginal sonography in women without history of preterm labor and at the gestational age of 21-24 wk.

**Table I:** Descriptive statistics of duration of pregnancy, the number of preterm and term labors, and age categories regarding cervical length

**Cervical length (mm)**	**Count**	**Duration of pregnancy** **(mean** **± ** **SD) weeks**	**Number of term labors**				**Number of preterm labors**		
			**>20 years old**	**≤20 years old**			**>20 years old**		**≤20 years old**
11- 15	6	33.16 ± 3.6	0	1		0		5	
16- 20	10	35.75 ± 1.4	0	2		1		7	
21- 25	30	36.98 ± 1.1	4	11		0		15	
26- 30	58	38.1 ± 1.08	4	46		1		7	
≥31	346	39.2 ± 1.1	25	310		0		11	

**Table II:** Evaluation of SPTD screening in women without history of preterm labor and at the gestational age of 21-24 weeks

**Cervical length (mm)**	**Sensitivity (%)**			**Specificity (%)**			**PPV (%)**			**NPV (%)**	
	**Point**	**CI**		**point**	**CI**		**point**	**CI**		**point**	**CI**
≥15	11.1	8.2-14		99.7	98.8-100		75	71-79		93.5	91.1-95.9
≥20	16.6	13.2-20		96	94.2-97.8		58	53.5-62.5		94.7	92.5-96.9
≥25	55.5	50.9-60.1		93.6	91.2-96		51	46.4-55.6		98.8	97.5-100
≥30	94.4	92.2-96.6		83.9	80.4-87.4		30.6	26.4-34.8		99.3	98.4-100

SPTD: Spontaneous preterm delivery.                     CI: Confidence Interval.

PPV: Positive predictive Value.                               NPV: Negative predictive Value.

## Discussion

The results of the present study showed significant relationship of cervical length at gestational age of 21-24wk with duration of pregnancy and preterm labor risk (p=0.001). Mean cervical length in participants with preterm labor was estimated to be 24.3mm which is approximately close to the estimated value in Imas *et al*, Berghella *et al* and Heath *et al* studies, but differs with 32mm reported in Farshchian study (24, 30-31). This finding is evident of the importance of cervical lengths of other than 25mm. The difference between the results of our study with Farshchian study can be attributed to the difference in the techniques of measuring cervical length.

In the present study compared to Mahshidian *et al* study, the percentage of SPTD cases was higher (approximately 10% vs. 3.5%), while percentage of positive funneling cases (1.3% approximately) was lower (10). In the mentioned study, participants were high risk women for preterm labor that can explain higher percent of funneling in them, but in regard to the reason of lower incidence of preterm labor in this group, it is necessary to pay attention to the borderline pregnancy durations for determining preterm labor in the two studies. According to the results of other studies mentioned in the introduction, the results of both Mahshidian *et al* study and the present study seem to be logic (21).

Based on the present study, funneling has significant role in decreasing cervical length and increasing the incidence of SPTD (p=0.001). This factor showed severe statistical relationship with preterm labor before 35^th^ week of gestation in Mahshidian *et al* study too, but its relationship with preterm labor before 37^th^ week of gestation has been unclear (21). This may be due to higher rate of preterm labor in lower gestational ages in the mentioned study as compared to the present study. Comparison of sample size in the two studies makes the consistency of the results of the present study more acceptable. Considering lower mean cervical length in women with history of SPTD compared to those without this history and significant relationship of cervical length with preterm labor incidence, the significance of difference in mean cervical length between the two groups of with and without history of SPTD can be well explained. Even though, attention to the disturbing factors such as causes of previous preterm labors can be of a great help in any decision making.

Maternal educational level and age had no effect on cervical length in the two groups of with and without preterm labor. This finding can guaranty the results of the present study in relation to the disturbing effects resulted from these factors. In spite of this, participants with preterm labor had jobs with significantly higher risk. Even though, small number of participants with difficult jobs in the present study prevented the possibility of exact conclusion and this issue can be investigated in future studies.

Although in table II, 5-mm cervical length classification has been presented for more exact evaluation of the results, according to the results of other studies, cervical length of 25mm can be considered as a cut off point for the screening of SPTD. In this level, half of preterm labors can be predicted and approximately 95% of normal individuals can be identified. Comparison of the results of the present study (with cutoff point of 20mm) with the results of Mahshidian *et al* study (with the cutoff point of 18mm) shows high agreement of the two studies (21). According to this, the reliability of SPTD screening based on cervical length is acceptable. Although the results of the present study are in line with the results of previous studies and confirm the efficacy of cervical length measurement in predicting preterm labor, simultaneous presence of affecting factors have not been analyzed statistically and it can be mentioned as one of the shortcomings of this study. 

## Conclusion

According to the present study, the incidence rate of spontaneous preterm labor before the 37^th^ week of gestation is approximately 10% and it reaches to approximately 3-4% before the 35^th^ week. Cervical length measured in transvaginal sonography has acceptable consistency for screening and early diagnosis of spontaneous preterm deliveries in low risk women. Even so, more comprehensive studies with considering simultaneous effects of risk factors are required for suggesting this screening program into the health care system of the country.
